# The Impact and Clinical Prediction of Hyperglycemia During Parenteral Nutrition for Nondiabetic Patients After Gastrectomy for Gastric Cancer

**DOI:** 10.3389/fnut.2022.807841

**Published:** 2022-02-14

**Authors:** Ning Lan, Xiaohua Chen, Ying Lu, Yujie Zhou, Fei Kong, Yining Zhao, Fuzhi Jiao, Lin Zhang, Wenzhen Yuan

**Affiliations:** ^1^The First School of Clinical Medicine, Lanzhou University, Lanzhou, China; ^2^Department of Radiation Oncology, The First Hospital of Lanzhou University, Lanzhou, China; ^3^Center for Family Medicine and Integrative Health Care, Beijing United Family Hospital, Beijing, China; ^4^Department of Surgical Oncology, The First Hospital of Lanzhou University, Lanzhou, China

**Keywords:** gastrectomy, hyperglycemia, complication, insulin, glycemic fluctuation

## Abstract

**Background and Purpose:**

Hyperglycemia (HG) is associated with increased postoperative complications. This study aims to evaluate the effect of HG during supplemental parenteral nutrition (SPN) on short-term prognosis in non-diabetic patients undergoing gastrectomy for cancer and to analyse the risk factors and prevention methods for HG.

**Methods:**

A total of 359 patients were divided into three groups according to blood glucose (BG) during SPN: normoglycemic patients ( ≤ 125 mg/dL), mild HG (125~200 mg/dL), and severe HG (>200 mg/dL). The effect of BG on postoperative short-term outcomes was analyzed. Multivariate regression was performed to investigate influencing factors for severe HG. The safety and efficacy of insulin addition to total nutrient admixture (TNA) for the prevention and management of HG were assessed by propensity score matching (PSM). In addition, regression analysis was performed in the noninsulin group to investigate the predictive factors of severe HG, and a nomogram was plotted.

**Results:**

The postoperative complication rate was 18.9%, but it was significantly higher in patients with severe HG than in mild HG and normoglycemic patients (25.2, 15.0, and 10.0%, respectively, *p* < 0.05). Multivariate logistic regression analysis showed that anemia, myosteatosis, higher postoperative capillary blood glucose (CBG) before TNA infusion, and insulin in the TNA were independent influencing factors for severe HG. Based on the above factors, 75 pairs of patients (insulin group and non-insulin group) with comparable baseline data were successfully matched by PSM. The HG incidence and the glycemic fluctuation were significantly improved through 1 U insulin/6 g glucose (1/6 scheme) to TNA. A nomogram containing hemoglobin, skeletal muscle radiodensity, pre-SPN CBG, and pTNM stage with good predictive efficacy (C-index: 0.750) was constructed based on the noninsulin group.

**Conclusion:**

Poor postoperative glycemic control was related to worse outcomes in non-diabetic patients undergoing gastrectomy for cancer. Pre-operative anemia, myosteatosis, and high postoperative CBG before TNA infusion are risk factors for severe HG. Insulin in TNA can improve the blood glucose control of patients. Our proposed nomogram rendered an individualized predictive tool for HG during SPN, which helps screen high-risk patients requiring insulin therapy. Future studies with larger samples are needed to develop a complete insulin application protocol for SPN.

## Introduction

Hyperglycemia (HG) is a common complication in hospitalized patients, occurring in 46% of intensive care unit (ICU) patients and 32% of non-ICU patients (1). Poor perioperative blood glucose (BG) control is closely associated with increased postoperative complications and mortality in colorectal, cardiac, and neurosurgery (2–7).

In 2017, Claudio Fiorillo et al. reviewed 173 non-diabetic patients who underwent gastrectomy and found that postoperative HG (BG>125 mg/dL) was a risk factor for higher postoperative mortality and complication rates (8), and postoperative HG was independently associated with decreased overall survival (OS) and disease-free survival (DFS) (9). However, the patients in this study did not receive any enteral nutrition (EN) within 72 h after surgery, and only 1,200 kcal calories per day were provided by infusion of normal saline and carbohydrates, which obeyed the nutritional treatment model under the concept of enhanced recovery after surgery (ERAS) (10–12).

Gastric cancer (GC) patients in China account for approximately 50% of the world (13) and are often accompanied by malnutrition (14). Therefore, perioperative nutritional treatment should be given to patients with nutritional risk screening (NRS-2002) ≥3 scores, which combines EN and supplemental parenteral nutrition (SPN). Data showed that the HG of EN patients was as high as 30%, and parenteral nutrition (PN) patients accounted for more than half (15). The American Society for Parenteral and Enteral Nutrition (ASPEN) recommends a BG target of 140~180 mg/dL for PN patients (16). However, insufficient attention has been given to the “hospital-related hyperglycemia” of non-diabetic patients in non-ICU situations due to the lack of monitoring equipment, nursing staff, etc.

This study aims to evaluate the current situation of HG for patients who need SPN after gastrectomy in a high-incidence area of GC in China. First, the effects of HG on short-term postoperative complications were analyzed. Subsequently, the independent influencing factors of severe HG were analyzed by multivariate logistic regression in all patients and noninsulin group, and a nomogram prediction model was constructed to help screen high-risk patients with HG during SPN.

## Materials and Methods

### Retrospective Cohort and Case-Control Study

#### Patients

There were 445 patients who underwent gastrectomy for cancer in the Department of Oncology, the First Hospital of Lanzhou University, between March 2017 and June 2021. The inclusion criteria included patients who (1) were 18–80 years old; (2) histologically confirmed gastric cancer; (3) radical gastrectomy performed with D2 lymphadenectomy; and (4) nutritional risk with nutritional risk screening (NRS 2002) scored ≥3. The exclusion criteria were as follows: (1) a known diagnosis of diabetes mellitus (DM); (2) admission random venous plasma glucose (VPG) >200 mg/dL; (3) other malignancies; (4) directly be transferred to direct transfer to the ICU after surgery; (5) HG occurrence after infection onset; and (6) incomplete preoperative CT data or postoperative BG data. Age, sex, BMI, inflammatory, nutritional status, and body composition index, surgical method, pathological stage, postoperative BG, and postoperative complications were collected. The study was performed following the Declaration of Helsinki and approved by the Ethics Committee of the First Hospital of Lanzhou University (Ethical approval number: LDYYLL-2021-272).

#### Inflammation, Nutritional Status, and Body Composition Index

Neutrophil-to-lymphocyte ratio (NLR) (17) = neutrophil count ( × 10^9^/L): lymphocyte count ( × 10^9^/L). Platelet-to-lymphocyte ratio (PLR) (17) = platelet count ( × 10^9^/L): lymphocyte count ( × 10^9^/L). Lymphocyte-to-monocyte ratio (LMR) (17): lymphocyte count ( × 10^9^/L): monocyte count ( × 10^9^/L). Prognostic nutritional index (PNI) (18): 10 × serum albumin (g/dL) + 0.005 × total lymphocyte count ( × 10^9^/L). Anemia is diagnosed when hemoglobin <120 g/L for men or hemoglobin <110 g/L for women. Hypoproteinemia is defined as albumin <35 g/L.

Patients underwent abdominal CT scans 1 month before the operation. A single slice CT image of the third lumbar vertebra (L3) was selected for body composition analysis. The images were analyzed by a single trained investigator using Slice-O-Matic 5.0 software (TomoVision, Montreal, Canada) to calculate the surface area of specific tissue types. Additionally, L3 skeletal muscle was evaluated using the tissue-specific HU thresholds of −29 to 150 (19). Entire muscle areas were normalized based on patient height and reported as the skeletal muscle index (SMI; cm^2^/m^2^). Patients were classified as sarcopenia according to established thresholds: L3 SMI <41 cm^2^/m^2^ for women; and <43 cm^2^/m^2^ for men with a BMI <25 and <53 cm^2^/m^2^ for men with a BMI ≥ 25 (20). The mean skeletal muscle density (SMD) within the L3 cross-section was recorded as a measure of myosteatosis, which was defined operationally as a mean SMD of <33 HU in patients with a BMI ≥25; and <41 HU in those with a BMI <25 across the axial orthogonal view (20).

#### Surgical Methods and Postoperative Nutritional Support

All patients underwent radical gastrectomy (proximal gastrectomy, distal gastrectomy, or total gastrectomy) with D2 lymphadenectomy. According to the American Joint Committee on Cancer 8th edition staging system, the postoperative pathological tumor–node–metastasis (TNM) stage was determined.

Patients were allowed sips of water or nasointestinal tube feeding of 5% glucose sodium chloride solution up to 300 mL from postoperative day 1 (POD1). When the patient does not have diarrhea, abdominal pain, abdominal distension, or vomiting, start a liquid diet or use enteral nutritional emulsion (Fresubin) for EN from POD2. EN is carried out in strict accordance with the principles of low concentration to high, liquid volume from less to more, and propulsion speed from slow to fast. All patients received SPN through TNA from POD1 until EN reached 60% of the patient's target energy. All TNAs were compounded in a timely manner in the hospital Pharmacy Intravenous Admixture Services Center.

#### BG Monitoring

The BG data of all recruited patients were recorded, including admission random VPG, capillary blood glucose (CBG) after returning to the ward on the day of operation (recorded as pre-SPN CBG), POD1 VPG (at 07:00), and during SPN CBG (4 times daily at 06:00, 12:00, 18:00, 00:00). The highest pre-SPN CBG was recorded as pre-SPN CBGmax. The first CBG at POD1 06:00 was recorded as POD1 CBG1. To reduce the differences caused by surgical stress in patients, this study analyzed only SPN BG in the first 3 days. Patients were divided into three groups according to CBG during SPN: normoglycemic patients (all CBG ≤ 125 mg/dL), mild HG (125 < CBG ≤ 200 mg/dL), and severe HG (CBG >200 mg/dL, detected for more than twice).

#### Postoperative Short-Term Complications

Total postoperative complications were defined as complications of grade II or higher according to the Clavien–Dindo (21) classification within 30 days after gastrectomy. Grades III to V complications were defined as severe complications. In addition, the postoperative comprehensive complications index (CCI) (22, 23), which contains grades I to V, was calculated. Postoperative mortality was defined as death within 30 days of surgery. Because prophylactic antibiotics were commonly prescribed for all patients, only prolonged antibiotic use or antibiotic change due to infection were included in this study.

#### Propensity Score Matching

For the comparison of the function of insulin in TNA, propensity score matching (PSM) was performed to match patients in the insulin and noninsulin groups. PSM was generated using a logistic regression model of the treatment on the following baseline covariates considered potential confounding factors: age, PNI, anemia, myosteatosis, type of reconstruction, pre-SPN CBGmax, POD1 CBG1, and glucose in TNA. We then conducted a 1:1 match between the insulin group and the noninsulin group. Optimal matching with a caliper size of 0.2 was used to avoid poor matches.

#### Indicators Reflecting Blood Glucose Control

Blood glucose control rate (BGCR): the percentage of 70–200 mg/dL of all BG test values in the total number of tests. Standard deviation (SD): means of SD of intraday BG. The largest amplitude of glycemic excursions (LAGE): means of the differences between the maximum and minimum BG values. Intraday coefficient of variation (CV): means of the ratios of the intraday glycemic standard deviation to the mean. Fasting blood glucose coefficient of variation (FBG-CV) (24): the ratio of the standard deviation of the mean of the glucose level at 06:00 AM per day during SPN. Hypoglycemia: The lowest CBG monitored during SPN infusion was <70 mg/dL.

#### Statistical Analysis

Statistical analysis was performed using commercially available software (version 26.0, SPSS, Inc., an IBM Company, Chicago, IL). Categorical data are presented as numbers (percentages). Continuous variables were tested for normality and are presented as the mean [±standard deviation (SD)] if normally distributed or as the median [±interquartile range (IQR)] if not normally distributed. For GroupWise comparisons, Student's *t*-test, Mann–Whitney U test, and Kruskal–Wallis test were used. Categorical data were compared using the χ2 test or Fisher's exact test. Paired sample nonparametric tests and McNemar χ2 tests were used for data analysis. Univariate and multivariate logistic regression analyses of the variables (forward conditional) were performed on clinicopathological parameters affecting postoperative BG, and R Version 4.0.4 was applied to plot the nomogram. α = 0.05 on both sides was taken as the test level, and *p* < 0.05 was considered statistically significant.

## Results

### Patient Characteristics

**Figure 1:**
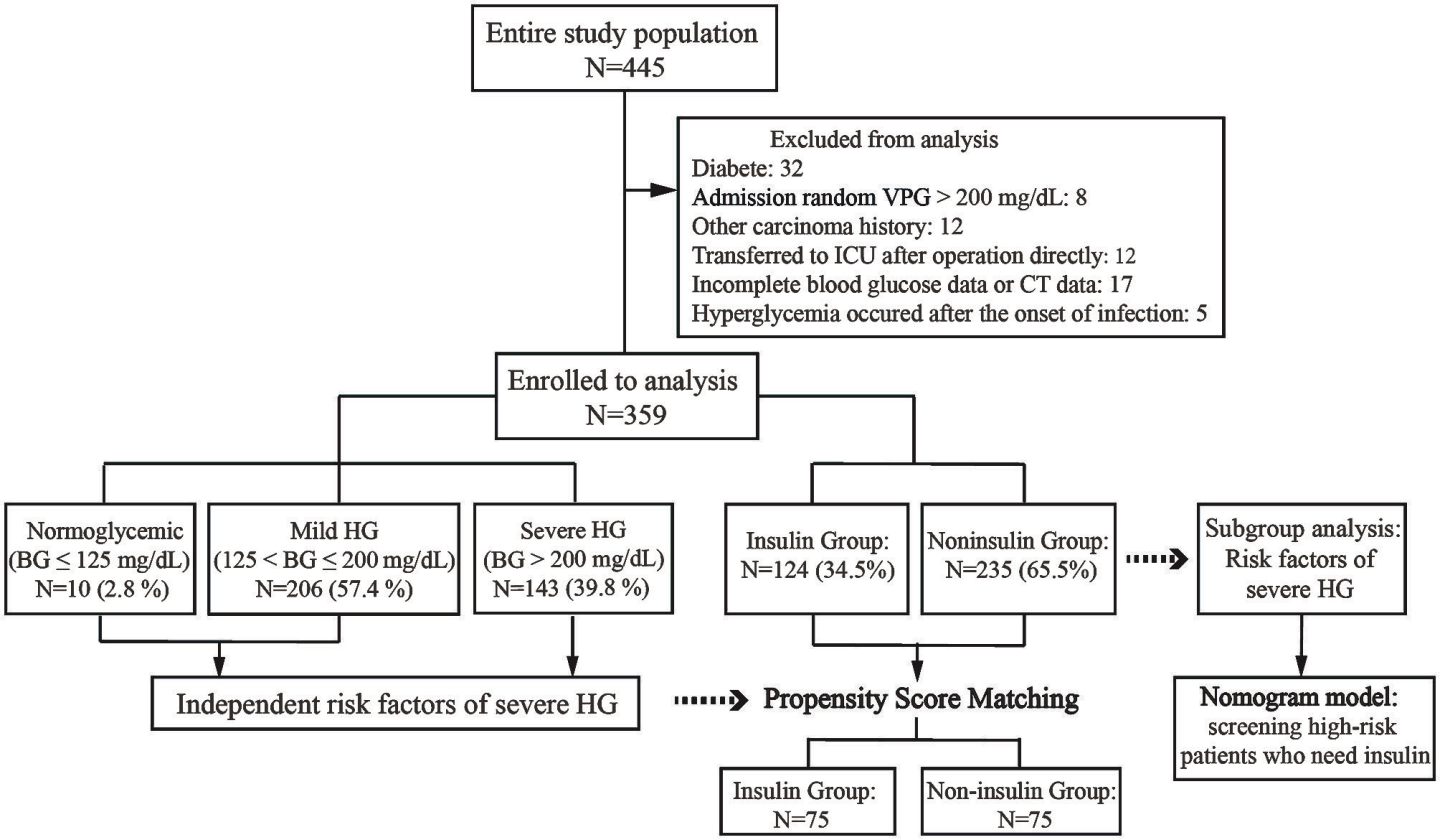
The flowchart of the study population selection. HG, hyperglycemia; BG, blood glucose.

A total of 445 gastrectomies for cancer were performed at our institution during the study period. Of 445 patients, 40 (9.0%) patients had preexisting DM or random VPG>200 mg/dL, 12 (2.7%) patients had a history of malignant tumor origins from other tissue, 12 (2.7%) patients were directly transferred to the ICU after the operation, and 17 (3.8%) patients had incomplete oerioperative data. Finally, 359 patients were included (Figure 1). The patients' mean age was 58.7 years, and BMI was 22.07 kg/m^2^. Among them, 129 (35.9%) patients received neoadjuvant chemotherapy (NAC), 239 (66.6%) patients underwent open surgery, and 120 patients (33.4%) underwent laparoscopic surgery. Proximal gastrectomy was performed in 4 patients (1.1%), distal gastrectomy in 187 patients (52.1%), and total gastrectomy in 168 patients (46.8%).

### The SPN HG's Contribution on Short-Term Outcomes

According to the CBG during SPN, the patients were divided into three groups: 10 (2.8%) normoglycemic patients, 206 (57.4%) patients with mild HG, and 143 (39.8%) patients with severe HG (Table 1). The maximum CBG during SPN in mild HG patients was 193 (175, 212.5) mg/dL, while that in severe HG patients was 257 (234, 286) mg/dL. The average CBG during SPN in severe HG patients was 164 (154, 180) mg/dL, which was significantly higher than that in patients with mild HG [133 (123, 144) mg/dL] and normoglycemic patients [102 (89.5, 109.8) mg/dL]. Postoperative outcomes among the three groups, postoperative complications and CCI were compared (Table 1). The total postoperative complication rate was 18.9% (68/359), which was significantly higher in patients with severe HG than in mild HG and normoglycemic patients (25.2, 15.0, and 10.0%, respectively, *p* = 0.045). The CCI of patients with severe HG was significantly higher than that of patients with mild HG and normoglycemic patients [8.7 (0, 20.9), 0 (0, 8.7), and 0 (0, 2.2)], *p* = 0.018, respectively]. The rate of severe complications in the three groups was zero in normoglycemic patients, 3.4% in mild HG, and 5.6% in severe HG (*p* = 0.625). Even if postoperative severe complications and mortality were more frequent in patients with higher CBG, no significant difference was detected among the three groups. All complications are listed in (Table 1).

**Table 1:** Perioperative outcomes.

	**Overall (*N* = 359)**	**NG (*N* = 10)**	**Mild HG (*N* = 206)**	**Severe HG (*N* = 143)**	***p*-value**
CCI, median (IQR)^†^	0.0 (0.0, 8.7)	0.0 (0.0, 2.2)	0.0 (0.0, 8.7)	8.7 (0.0, 20.9)	0.018^*^
**Degree of complications** ^‡^					
≥Grade II, *n* (%)	68 (18.9)	1 (10.0)	31 (15.0)	36 (25.2)	0.045^*^
≥Grade III, *n* (%)	15 (4.2)	0	7 (3.4)	8 (5.6)	0.625
Grade V, *n* (%)	3 (1.1)	0	2 (1.0)	1 (0.7)	1.000
**Summary of all complications**					
**Grade II**					
Infectious complications^§^	38	1	16	21	
Blood transfusion	28	0	14	14	
Bowel obstruction	1	0	1	0	
**Grade III**					
Anastomotic leak	3	0	2	1	
Pelvic abscess	2	0	0	2	
Wound dehiscence	5	0	2	3	
Intraabdominal hemorrhage	2	0	1	1	
Bowel obstruction	2	0	1	1	
**Grade IV**					
Respiratory failure	4	0	3	1	
Circulatory failure	2	0	1	1	

*NG, normoglycemic; HG, hyperglycemia; CCI, comprehensive complications index; IQR, interquartile range*.

* *Statistically significant*.

† *The comprehensive complication index (CCI) is based on the complication grading by Clavien-Dindo classification and implements every occurred complication, which can be calculated on https://www.assessurgery.com/about_cci-calculator/. The overall morbidity is reflected on a scale from 0 (no complication) to 100 (death)*.

‡ *The highest grade complications for each patient based on Clavien-Dindo classification*.

§ *For the same patient, infection at different sites was calculated as one case*.

### Risk Factors During SPN HG

To analyse the risk factors for severe HG during SPN, the clinicopathological characteristics of patients with severe HG and other patients were compared in this study. Univariate regression analysis showed that age, PNI, anemia, myosteatosis, type of reconstruction, pre-SPN CBGmax, POD1 CBG1, insulin and glucose in the TNA were closely associated with severe HG during SPN (Table 2). Multivariate logistic regression analysis of the variables (forward conditional) showed that anemia, myosteatosis, pre-SPN CBGmax, POD1 CBG1, and insulin in TNA were independent risk factors affecting HG. Among them, insulin was an independent protective factor. The incidence of severe HG increased significantly after insulin infusion. (OR = 5.334, 95% CI: 2.984~9.533, *p* = 0.000) (Table 2).

**Table 2:** Univariate and multivariate analysis for severe HG during SPN in all patients.

	**NG+Mild HG (*n* = 216)**	**Severe HG (*n* = 143)**	***p*-value**	**Univariate analysis**			**Multivariate analysis**		
				**OR**	**95%CI**	***p***	**OR**	**95%CI**	***p*-value**
Age ≥ 65 years, *n* (%)	58 (26.9)	59 (41.3)	0.004^*^	1.913	1.222–2.997	0.005^*^			
Female, *n* (%)	43 (19.9)	38 (26.6)	0.139	1.456	0.884–2.399	0.140			
BMI, $\bar{\text{x}}$ ± SD, kg/m^2^	22.0 ± 3.1	22.1 ± 2.6	0.757	1.001	0.941–1.087	0.756			
NAC, *n* (%)	70 (32.4)	59 (41.3)	0.087	1.465	0.945–2.271	0.088			
Admission VPG, m (IQR), mg/dL	90.7 (83.3, 100.2)	90.7 (84.4, 102.2)	0.403	1.009	0.997–1.020	0.133			
NLR, median (IQR)	2.1 (1.5, 2.9)	2.1 (1.5, 3.5)	0.394	1.058	0.975–1.148	0.179			
PLR, median (IQR)	132.1 (91.0, 173.9)	120.5 (87.5, 187.3)	0.785	1.002	0.999–1.004	0.167			
LMR, median (IQR)	4.1 (3.2, 5.6)	3.8 (2.8, 5.1)	0.055	0.917	0.827–1.017	0.101			
PNI, median (IQR)	434.5 (405.0, 458.5)	429.0 (396.0, 451.0)	0.061	0.995	0.990–1.000	0.038^*^			
Hypoproteinemia, *n* (%)	7 (3.2)	8 (5.6)	0.275	1.769	0.627–4.992	0.281			
Anemia, *n* (%)	39 (18.1)	56 (39.2)	0.000^*^	2.921	1.803–4.734	0.000^*^	2.467	1.429–4.262	0.001^*^
Sarcopenic, *n* (%)	79 (36.6)	58 (40.6)	0.447	1.183	0.767–1.826	0.447			
Myosteatosis, *n* (%)	42 (19.4)	56 (39.2)	0.000^*^	2.667	1.657–4.291	0.000^*^	1.931	1.122–3.323	0.018^*^
Operation approach, *n* (%)			0.061						
Open	152 (70.4)	87 (60.8)		1					
Laparoscopic	64 (29.6)	56 (39.2)		1.529	0.980–2.386	0.062			
Surgery type, *n* (%)			0.454						
Proximal gastrectomy	3 (1.4)	1 (0.7)		1					
Distal gastrectomy	118 (54.6)	69 (48.3)		1.754	0.179–17.195	0.629			
Total gastrectomy	95 (44.0)	73 (51.0)		2.305	0.235–22.620	0.473			
Type of reconstruction, *n* (%)			0.000^*^						
Billroth I	17 (7.9)	2 (1.4)		1					
Billroth II	48 (22.2)	16 (11.2)		2.833	0.589–13.627	0.194			
Roux-en-Y	151 (69.9)	125 (87.4)		7.036	1.595–31.041	0.010^*^			
pTNM stage, n (%)			0.231						
I	71 (32.9)	42 (29.4)		1					
II	49 (22.7)	44 (30.8)		1.518	0.869–2.652	0.143			
III	96 (44.4)	57 (39.9)		1.004	0.607–1.660	0.988			
Pre-SPN CBGmax, median (IQR), mg/dL	148.0 (128.0, 180.0)	164.0 (138.0, 198.0)	0.001^*^	1.007	1.002–1.012	0.003^*^	1.006	1.000–1.011	0.035^*^
POD1 CBG1, median (IQR), mg/dL	113.4 (100.8, 127.8)	120.6 (106.7, 140.0)	0.002^*^	1.016	1.007–1.025	0.001^*^	1.015	1.005–1.026	0.005^*^
POD1 VPG, median (IQR), mg/dL	107.6 (74.9, 120.6)	107.3 (97.0, 124.8)	0.312	1.008	1.000–1.016	0.064			
No insulin in TNA, *n* (%)	118 (54.6)	117 (81.8)	0.000^*^	3.737	2.262–6.176	0.000^*^	5.334	2.984–9.533	0.000^*^
Glucose in TNA, median (IQR), g	250 (200, 250)	250 (200, 250)	0.000^*^	1.010	1.003–1.017	0.007^*^			

*NG, normoglycemic; HG, hyperglycemia; NAC, neoadjuvant chemotherapy; VPG, venous plasma glucose; IQR, interquartile range; NLR, neutrophil-to-lymphocyte ratio; PLR, platelet-to-lymphocyte ratio; LMR, lymphocyte-to-monocyte ratio; PNI, prognostic nutritional index; Pre-SPN CBGmax, the highest capillary blood glucose before supplemental parenteral nutrition; POD1 CBG1, the capillary blood glucose at 06:00 of postoperative day 1; POD1 VPG, venous plasma glucose of postoperative day 1; TNA, total nutrient admixture*.

* *Statistically significant*.

### The Safety and Efficacy of Insulin Addition to TNA

In this study, 235 patients were not treated with insulin, and 124 patients received insulin-containing TNA due to different perceptions of PN from surgeons. We found that the proportion of insulin was 1 U insulin/6 g glucose (1/6 scheme) in the TNA of 91.9% (114/124) patients in the insulin group. In addition, the proportion of insulin was 1 U/4 g in 2 patients, 1 U/5 g in 6 patients, 1 U/8 g in 1 patient, and 1 U/10 g in 1 patient. To assess the safety and efficacy of the 1/6 scheme, we performed PSM analysis, including 8 covariates (age, PNI, anemia, myosteatosis, type of reconstruction, pre-SPN CBGmax, POD1 CPG1, glucose in TNA).

Seventy-five pairs of patients were successfully matched and defined as the insulin group and non-insulin group. There was no significant difference in the clinicopathological data apart from the surgical approach (Supplementary Table 1). The results showed that the incidence of HG in the insulin group was 93.3% (70/75) and that in the non-insulin group was 100% (*p* < 0.05). During SPN, the mean and maximum CBG concentrations in the insulin group were significantly lower than those in the non-insulin group (*p* < 0.05). The BGCR of patients in the insulin group was 91.7 (83.3, 100) %, which was higher than that of patients in the non-insulin group [90.9 (75.0, 100) %, *p* = 0.011] (Table 3). In addition, glycemic fluctuation and insulin-related hypoglycemia are risk factors for increased postoperative complications and mortality (25, 26). The intraday glycemic fluctuation (SD, LAGE, and CV) and interday glycemic fluctuation (FBG-CV) (27–29) were evaluated (Table 3), indicating that the fluctuation of the patients in the insulin group was less than that in the non-insulin group. Except for FBG-CV, the others were statistically significant. The results showed that insulin in PN has positive significance to glycemic control. In addition, the McNemar χ2 test was performed on the occurrence of hypoglycemic events to evaluate the safety of this insulin dosage, indicating that the 1/6 scheme increased the incidence of hypoglycemia in patients (*p* = 0.039). However, the postoperative complication rates and CCI were not significantly different between the groups (Table 3).

**Table 3:** The effect of insulin on 75 pairs of patients.

	**Non-insulin (*N* = 75)**	**Insulin (*N* = 75)**	***p-value***
**Degree of total BG control**			
NG, *n* (%)	0	5 (6.7)	
Mild HG, *n* (%)	39 (52.0)	54 (72.0)	0.032^*^
Severe HG, *n* (%)	36 (48.0)	16 (21.3)	0.003^*^
BG Mean, median (IQR), mg/dL	150.4 (130.5, 164.6)	134.9 (121.9, 151.2)	0.001^*^
BG Maximum, median (IQR), mg/dL	217.8 (187.2, 264.6)	194.4 (165.6, 226.8)	0.001^*^
BGCR, median (IQR), %	90.9 (75.0, 100)	91.7 (83.3, 100)	0.011^*^
**BG fluctuation**			
SD, median (IQR), mg/dL	41.4 (29.9, 50.8)	29.5 (23.9, 39.8)	0.000^*^
LAGE, median (IQR), mg/dL	120.6 (99, 162.0)	100.8 (75.6, 129.6)	0.001^*^
CV, median (IQR), %	26.7 (21.5, 33.2)	22.7 (18.9, 27.7)	0.001^*^
FBG-CV, median (IQR), %	14.3 (7.8, 23.0)	13.0 (7.7, 22.0)	0.750
**Safety of insulin in TNA**			
Hypoglycemia, *n* (%)	2 (2.7)	10 (13.3)	0.039^*^
**Short-term outcomes**			
CCI, median (IQR)	0 (0, 8.7)	0 (0, 12.3)	0.069
≥Grade II, *n* (%)	8 (10.7)	18 (24.0)	0.052
≥Grade III, *n* (%)	1 (1.3)	3 (4.0)	0.625

*BG, blood glucose; NG, normoglycemic; HG, hyperglycemia; BG, blood glucose; IQR, interquartile range; BGCR, blood glucose control rate; SD, standard deviation; LAGE, largest amplitude of glycemic excursions; CV, coefficient of variation; FBG-CV, fasting blood glucose coefficient of variation; CCI, comprehensive complications index*.

* *Statistically significant*.

### The Screen of High-Risk Patients With HG During SPN

**Figure 2:**
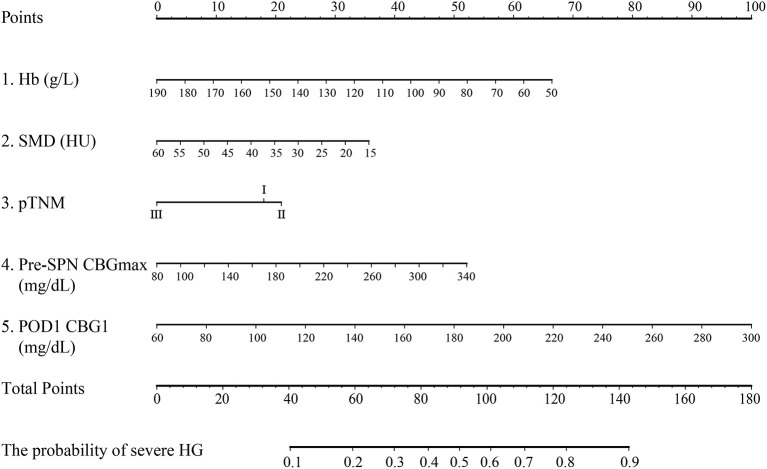
Nomogram predicting HG during SPN for nondiabetic patients after gastrectomy for gastric cancer. Hb, hemoglobin; SMD, skeletal muscle density; pre-SPN CBGmax: the highest postoperative capillary blood glucose before supplemental parenteral nutrition; POD1 CBG1, the capillary blood glucose at postoperative day 1 06:00.

In this study, all patients in the noninsulin group suffered HG, and 49.8% (117/235) patients had severe HG. In contrast, 71.0% of patients in the insulin group experienced mild HG. The rates of severe HG and normoglycemia were 21.0 and 8.1%, respectively. Moreover, with or without insulin, some patients suffered hypoglycemia, with an incidence of 12.9% (16/124) in the insulin group and 0.4% (14/235) in the noninsulin group. The above results suggest that for non-diabetic patients with SPN after gastrectomy, the incidence of HG is very high and should be given sufficient attention in the clinic. Hypoglycemia caused by insulin therapy should also be avoided. There is an urgent need to propose a simple tool to help screen high-risk patients with HG and provide preventive insulin treatment. Regression analysis was performed on all patients in the noninsulin group. The results show that anemia, myosteatosis, pTNM stage, pre-SPN CBGmax, and POD1 CBG1 are high-risk factors for severe HG (Table 4). In order to simplify clinical application, the nomogram was plotted and has moderate accuracy for severe HG prediction (C-index = 0.750) (Figure 2).

**Table 4:** Univariate and multivariate analysis for severe HG during SPN of noninsulin group (*N* = 235).

	**Univariate regression analysis**			**Multivariate regression analysis**		
	**OR**	**95%CI**	***p-value***	**OR**	**95%CI**	***p-value***
Age ≥ 65 years, *n* (%)	2.061	(1.178–3.603)	0.011^*^			
Female, *n* (%)	1.291	(0.698–2.385)	0.415			
BMI, $\bar{\text{x}}$ ± SD, kg/m^2^	1.012	(0.924–1.109)	0.793			
NAC, *n* (%)	1.312	(0.770–2.238)	0.318			
Admission VPG, median (IQR), mg/dL	1.005	(0.991–1.019)	0.486			
NLR, median (IQR)	1.138	(0.977–1.326)	0.096			
PLR, median (IQR)	1.003	(1.000–1.006)	0.072			
LMR, median (IQR)	0.884	(0.783–0.998)	0.047^*^			
PNI, median (IQR)	0.995	(0.990–1.001)	0.119			
Hypoproteinemia, *n* (%)	4.257	(0.884–20.490)	0.071			
Anemia, *n* (%)	4.281	(2.277–8.051)	0.000^*^	3.754	(1.836–7.674)	0.000^*^
Sarcopenic, *n* (%)	1.304	(0.770–2.207)	0.323			
Myosteatosis, *n* (%)	3.144	(1.741–5.679)	0.000^*^	2.241	(1.140–4.404)	0.019^*^
**Operation approach**, ***n*** **(%)**
Open	1					
Laparoscopic	0.949	(0.569–1.584)	0.842			
**Surgery type**, ***n*** **(%)**
Proximal gastrectomy						
Distal gastrectomy	1					
Total gastrectomy	0.919	(0.551–1.532)	0.745			
**Type of reconstruction**, ***n*** **(%)**
Billroth I	1					
Billroth II	0.000	0.000	1.000			
Roux-en-Y	0.000	0.000	1.000			
**pTNM Stage**, ***n*** **(%)**
I	1.302	(0.705–2.407)	0.399	2.314	(1.126–4.754)	0.022^*^
II	1.881	(1.000–3.537)	0.050	2.535	(1.212–5.302)	0.013^*^
III	1			1		
Pre-SPN CBGmax, median (IQR), mg/dL	1.012	1.006–1.019	0.000^*^	1.010	(1.002–1.017)	0.012^*^
POD1 CBG1, median (IQR), mg/dL	1.025	(1.011–1.038)	0.000^*^	1.018	(1.004–1.033)	0.014^*^
POD1 VPG, median (IQR), mg/dL	1.008	(0.997–1.019)	0.148			
Glucose in TNA, median (IQR), g	1.002	(0.994–1.010)	0.612			

*NAC, neoadjuvant chemotherapy; VPG, venous plasma glucose; IQR, interquartile range; NLR, neutrophil-to-lymphocyte ratio; PLR, platelet-to-lymphocyte ratio; LMR, lymphocyte-to-monocyte ratio; PNI, prognostic nutritional index; Pre-SPN CBGmax, the highest capillary blood glucose before supplemental parenteral nutrition; POD1 CBG1, the capillary blood glucose at postoperative day 1 06:00; POD1 VPG, venous plasma glucose of postoperative day 1; TNA, total nutrient admixture*.

* *Statistically significant*.

## Discussion

Postoperative HG is positively correlated with postoperative infection and sepsis in patients with or without diabetes (30–32). Uncontrolled HG is associated with total complications and mortality after general surgery (32, 33). The results confirm the idea that HG during SPN is associated with a higher incidence of complications in non-diabetic patients after gastrectomy for gastric cancer. Anemia, myosteatosis, higher postoperative CBGs before TNA infusion, and insulin in the TNA are independent risk factors for postoperative severe HG. In addition, the 1/6 scheme has a good effect on reducing postoperative HG and glycemic fluctuation with the risk of increased hypoglycemia. The constructed nomogram can help screen high-risk patients who urgently need insulin therapy.

Claudio Fiorillo et al. reviewed non-diabetic patients undergoing selective gastric cancer surgery, and 55.5% of them had HG after surgery. The postoperative complication rates of patients with severe HG (BG>200 mg/dL), mild HG (BG between 125 and 200 mg/dL) and normoglycemic patients (BG ≤ 125 mg/dL) were 63.6, 30.6, and 13%, respectively (*p* < 0.001) (8). Severe HG is a risk factor for complications and mortality within 30 days after the operation, and its OR value is greater than the TNM stage and intraoperative blood transfusion (8). The results of this study show that 97.2% (349/359) of patients suffer HG during SPN in the first 3 days, and 39.8% (143/359) of patients suffer severe HG, which may be related to the nutritional treatment our patients received. The glucose load and administration rate of TNA are important factors affecting the occurrence of HG (15, 34). The incidence of postoperative complications in severe HG patients was significantly higher than that in mild HG and normoglycemic patients (25.2, 15.0, and 10.0%, respectively, *p* < 0.05). The results showed that patients' risk of postoperative complications, severe complications, and mortality were parallel to the degree of elevated BG, consistent with the findings of Claudio Fiorillo et al.

Steve Kwon et al. found that perioperative and postoperative HG in general surgery patients with and without diabetes was associated with a nearly 2-fold higher risk of infection, in-hospital mortality, and operative complications (33). However, patients with HG who received insulin were at no greater risk than those with normal BG (33). Patients without a history of diabetes who experienced HG had the greatest risk of infection. Therefore, it is necessary to regularly use insulin to prevent and treat “hospital-related hyperglycemia” in non-diabetic patients who need surgery (35). Levitan et al. evaluated 1,034 adults consecutively hospitalized without a diagnosis of diabetes at the time of admission. Among them, HG occurred in 37.5% of medical patients and 33% of surgical patients. Fifty-four percent received insulin therapy, and 59% received bedside glucose monitoring in all patients (36). In addition, insulin has been found to improve skeletal muscle protein metabolism in cancer patients after major surgery (37). The anti-inflammatory effect of insulin on patients with elevated C-reactive protein (CRP) and the role of preventing arrhythmia by reducing free fatty acids have also been confirmed (38, 39). Continuous intravenous infusion of insulin or direct addition to TNA are effective methods to control HG during PN (34, 40). In consideration of the adsorption effect of plastic containers on drugs, intravenous infusion of insulin alone is recommended in the guidelines (41). However, with the update of intravenous infusion materials in recent years, increasing studies have pointed out that adding insulin to TNA is a feasible and safe choice in line with physiological mechanisms (34, 40). Due to the lack of relevant research evidence, there is a short of consensus on the timing and dosage of insulin therapy for nondiabetic patients who currently need PN. Some studies suggest adding 0.05 U insulin/g glucose to TNA when BG>150 mg/dL is detected twice (42, 43). Then, insulin levels were increased in increments of 0.05–0.1 U/g dextrose to maintain BG within an acceptable range (<180 mg/dL). For those without a known history of diabetes but with two consecutive serum BG values>180 mg/dL, insulin was added at 0.5 U/g dextrose and increased as described above (44).

In China, the 2018 edition of consensus for parenteral nutrition solutions proposed that prophylactic insulin was not recommended for patients with normal BG who received PN (41). At the same time, other scholars have advocated that 1 U insulin/4~10 g glucose can be routinely added to TNA for nondiabetic patients (45, 46). In this study, 75 pairs of patients with or without insulin were obtained through PSM, reducing the observational study's confounding bias and selectivity bias (47). The results stated that the 1/6 scheme significantly improved glycemic control in patients. However, there was no significant difference in the incidence of complications between the insulin group and the non-insulin group, indicating that simply adding insulin in a fixed proportion can not optimize the BG control of patients. There is an urgent need for a simple and easy way to screen high-risk patients with postoperative severe HG.

In this study, a nomogram prediction model with five factors was established through regression analysis on the patients in the noninsulin group. In addition to the postoperative CBG before TNA infusion, we found that hemoglobin and SMD were important independent factors affecting the occurrence of severe HG. In recent years, increasing studies have focused on the independent clinical significance of myosteatosis diagnosed with SMD (48, 49). Myosteatosis is an ectopic fat bank that increases with age. Lipid deposition in skeletal muscle leads to the accumulation of lipid intermediates (diacylglycerol and ceramide) and destroys the insulin signaling pathway, leading to insulin resistance (IR) and the development of type 2 diabetes (48, 50, 51). In addition, researchers have found that there is a positive correlation between abdominal adipose tissue and the homeostasis model assessment of insulin resistance (HOMA-IR) in elderly white men without diabetes (52) and a negative correlation with insulin sensitivity (53). Moreover, anemia can produce IR, which has been proven to be one of the risk factors for stress HG after major abdominal surgery (54). Studies have shown that hemoglobin is an important predictor of low SMD (55), and their relationship further supports our results.

It is challenging for non-diabetic patients with PN to determine when to add what dose of insulin to achieve maximum glycemic control (44). Routine prophylactic administration of insulin is dangerous, leading to hypoglycemia and even death (40). In 2013, Kelly et al. found that DM patients, PN in the ICU, number of days of PN, and insulin in PN were strong predictors of hypoglycemia (43). However, this study did not address the risk factors for hypoglycemia in non-ICU non-diabetic patients with SPN. In this study, 75 paired patients showed that the 1/6 scheme increased the incidence of hypoglycemia. In addition, 6.0% (14/235) of the patients had a hypoglycemic event in the noninsulin group. Therefore, insulin prevention is controversial for a small number of patients. For these patients, pre-SPN CBG should be monitored immediately after the operation, and the need for preventive insulin treatment should be determined according to hemoglobin, SMD, and pTNM stages. It will be the direction of our future efforts to develop individualized insulin prevention programs that integrate the above factors to benefit more patients.

This study still has some limitations. (1) This study is a retrospective, single-center study. Therefore, it is necessary to conduct a further large-scale, multicenter investigation and prospective study to explore safer individualized PN insulin application programs. (2) Glycated hemoglobin (HbA1c) can better reflect the baseline BG status of patients and is not affected by diet or exercise in the short term. In this study, the included patients were not routinely tested for HbA1c, so an important indicator was missing in the risk factor analysis. (3) At present, the standardization and timeliness of glycemic control in PN for non-ICU patients are not enough. Whether to add insulin in TNA was determined by the doctor's experience. This may be a common procedure seen in many Chinese non-critical surgical departments, and most of the time, there is a lack of attention to possible SHG occurring in perioperative patients. As a result, some patients have not received ideal insulin treatment.

## Conclusion

In this study, HG was common during SPN after gastrectomy for gastric cancer in non-diabetic patients, and postoperative HG was associated with a higher complication rate. Patients' hemoglobin, SMD, pTNM stage, pre-SPN CBGmax, and POD1 CBG1 are strong predictors of severe HG, which should be monitored pre-SPN in detail. For some high-risk patients for HG, appropriate PN insulin should be given, and 1 U insulin/6 g glucose is a dose that can be referred to. Future studies with larger samples are needed to develop a complete SPN insulin application program after gastrectomy to achieve optimal glycemic control and improve the prognosis.

## Data Availability Statement

The raw data supporting the conclusions of this article will be made available by the authors, without undue reservation.

## Ethics Statement

The studies involving human participants were reviewed and approved by Ethics Committee of the First Hospital of Lanzhou University. Written informed consent for participation was not required for this study in accordance with the national legislation and the institutional requirements.

## Author Contributions

WY, NL, XC, YL, and LZ designed the study, involved in the data collection, analysis, and drafting of the manuscript. XC, YZhou, FK, YZhao, and FJ were involved in the design of the study, analysis of the data, and critically reviewed the manuscript. All authors read and approved the final manuscript.

## Funding

This work was supported by the Excellent Plan for Student Scientific Research Innovation Cultivation Project of Lanzhou University (20210060135).

## Conflict of Interest

The authors declare that the research was conducted in the absence of any commercial or financial relationships that could be construed as a potential conflict of interest.

## Publisher's Note

All claims expressed in this article are solely those of the authors and do not necessarily represent those of their affiliated organizations, or those of the publisher, the editors and the reviewers. Any product that may be evaluated in this article, or claim that may be made by its manufacturer, is not guaranteed or endorsed by the publisher.
