# Supplementary material for: The Impact and Clinical Prediction of Hyperglycemia During Parenteral Nutrition for Nondiabetic Patients After Gastrectomy for Gastric Cancer
Source: Front Nutr. 2022 Feb 14;9:807841. doi: 10.3389/fnut.2022.807841 (PMC8882839; doi:10.3389/fnut.2022.807841)
Supplement: Supplementary file 1 [file Table_1.docx]

**Supplement Table 1: Base clinical data of seventy-five pair patients.**

|  | Noninsulin (N=75) | Insulin (N=75) | *p-value* |
| --- | --- | --- | --- |
| Age ≥ 65 years, n (%) | 24 (32.0) | 24 (32.0) | 1.000 |
| Female, n (%) | 55 (73.3) | 59 (78.7) | 0.541 |
| BMI, ‾x ± SD, kg/m^2^ | 21.9 ± 2.4 | 21.9 ± 3.2 | 0.728 |
| NAC, n (%) | 19 (25.3) | 30 (40.0) | 0.126 |
| Admission VPG, median (IQR), mg/dL | 90.7 (84.2, 97.4) | 88.4 (83.0, 101.7) | 0.853 |
| NLR, median (IQR) | 2.0 (1.3, 3.1) | 2.0 (1.4, 2.8) | 0.792 |
| PLR, median (IQR) | 125.4 (91.6, 184.8) | 123.3 (81.5, 170.6) | 0.318 |
| LMR, median (IQR) | 4.2 (3.1, 5.5) | 4.0 (2.9, 5.2) | 0.540 |
| PNI, median (IQR) | 427.0 (396.0, 446.0) | 429.0 (404.0, 455.0) | 0.221 |
| Hypoproteinemia, n (%) | 6 (8.0) | 4 (5.3) | 0.754 |
| Anaemia, n (%) | 20 (26.7) | 17 (22.7) | 0.678 |
| Sarcopenic, n (%) | 32 (42.7) | 29 (38.7) | 0.749 |
| Myosteatosis, n (%) | 12 (16.0) | 16 (21.3) | 0.557 |
| Operation approach, n (%)  Open  Laparoscopic | 38 (50.7)  37 (49.3) | 68 (90.7)  7 (9.3) | 0.000 |
| Surgery type, n (%)  Proximal gastrectomy  Distal gastrectomy  Total gastrectomy | 0  41 (54.7)  34 (45.3) | 2 (2.7)  34 (45.3)  39 (52.0) | 0.531 |
| Type of reconstruction, n (%)  Billroth I  Billroth II  Roux-en-Y | 1 (1.3)  20 (26.7)  54 (72.0) | 5 (6.7)  21 (28.0)  49 (65.3) | 0.257 |
| pTNM stage, n (%)  I  II  III | 23 (30.7)  17 (22.7)  35 (46.7) | 25 (33.3)  17 (22.7)  33 (44.0) | 0.983 |
| Pre-SPN CBGmax, median (IQR), mg/dL | 156.0 (129.6, 192.6) | 165.6 (136.8, 190.8) | 0.624 |
| POD1 CBG1, median (IQR), mg/dL | 115.0 (103.0, 135.0) | 122.0 (106.0, 142.0) | 0.159 |
| POD1 VPG, median (IQR), mg/dL | 110.2 (97.4, 123.5) | 109.1 (98.7, 124.5) | 0.815 |
| Glucose in TNA, median (IQR), g | 250 (200, 250) | 200 (200, 250) | 0.053 |

NAC, neoadjuvant chemotherapy; VPG, venous plasma glucose; IQR, interquartile range; NLR, neutrophil-to-lymphocyte ratio; PLR, platelet-to-lymphocyte ratio; LMR, lymphocyte-to-monocyte ratio; PNI, prognostic nutritional index; Pre-SPN CBGmax, the highest capillary blood glucose before supplemental parenteral nutrition; POD1 CBG1, the capillary blood glucose at 06:00 of postoperative day 1; POD1 VPG, venous plasma glucose of postoperative day 1; TNA, total nutrient admixture.

* Statistically significant
